# Maternal Lutein and Zeaxanthin Concentrations in Relation to Offspring Visual Acuity at 3 Years of Age: The GUSTO Study

**DOI:** 10.3390/nu12020274

**Published:** 2020-01-21

**Authors:** Jun S. Lai, Vaishnavi O. Veetil, Carla Lanca, Bee Lan Lee, Keith M. Godfrey, Peter D. Gluckman, Lynette P. Shek, Fabian Yap, Kok Hian Tan, Yap Seng Chong, Choon Nam Ong, Cheryl S Ngo, Seang-Mei Saw, Mary F. F. Chong

**Affiliations:** 1 Singapore Institute for Clinical Sciences, Agency for Science and Technology Research, Singapore 117609, Singapore; lai_jun_shi@sics.a-star.edu.sg (J.S.L.); pd.gluckman@auckland.ac.nz (P.D.G.); paeshekl@nus.edu.sg (L.P.S.); obgcys@nus.edu.sg (Y.S.C.); 2 Department of Obstetrics and Gynaecology, Yong Loo Lin School of Medicine, National University of Singapore, Singapore 119228, Singapore; vaishnavi.ov@gmail.com; 3 Singapore Eye Research Institute, Singapore 169856, Singapore; carla.lanca@seri.com.sg (C.L.); saw.seang.mei@seri.com.sg (S.-M.S.); 4 Saw Swee Hock School of Public Health, National University of Singapore, Singapore 117549, Singapore; ephleebl@nus.edu.sg (B.L.L.); ephocn@nus.edu.sg (C.N.O.); 5 MRC Lifecourse Epidemiology Unit & NIHR Southampton Biomedical Research Centre, University of Southampton & University Hospital Southampton NHS Foundation Trust, Southampton SO16 6YD, UK; kmg@mrc.soton.ac.uk; 6 Liggins Institute, University of Auckland, Auckland 1023, New Zealand; 7 Department of Paediatrics, Yong Loo Lin School of Medicine, National University of Singapore and National University Health System, Singapore 119228, Singapore; 8 Duke-NUS Graduate Medical School, Lee Kong Chian School of Medicine, Singapore 169857, Singapore; fabian.yap.k.p@singhealth.com.sg; 9 Department of Paediatric Endocrinology, KK Women’s and Children’s Hospital, Singapore 229899, Singapore; 10 Department of Maternal Fetal Medicine, KK Women’s and Children’s Hospital, Singapore 229899, Singapore; tan.kok.hian@singhealth.com.sg; 11 Yong Loo Lin School of Medicine, National University of Singapore and National University Health System, Singapore 119228, Singapore; 12 Department of Ophthalmology, National University Hospital, Singapore 119074, Singapore; cherylngo@gmail.com

**Keywords:** lutein, zeaxanthin, pregnancy, visual acuity, child

## Abstract

Lutein and zeaxanthin play important roles in visual functions, but their influence on early visual development is unclear. We related maternal lutein and zeaxanthin concentrations during pregnancy to offspring visual acuity (VA) in 471 mother–child pairs from the Growing Up in Singapore Towards healthy Outcomes (GUSTO) cohort. Maternal concentrations of plasma lutein and zeaxanthin were determined at delivery. We measured uncorrected distance of VA in 3-year old children using a LEA Symbols chart; readings were converted to the logarithm of Minimum Angle of Resolution (logMAR), with >0.3 logMAR indicating poor VA. Associations were examined using linear or Poisson regression adjusted for confounders. The median (inter-quartile range) of maternal lutein and zeaxanthin concentrations were 0.13 (0.09, 0.18) and 0.09 (0.07, 0.12) µmol/L, respectively. A total of 126 children had poor VA. The highest tertile of maternal zeaxanthin concentration was associated with 38% lower likelihood of poor VA in children (95% CI: 0.42, 0.93, *p*-Trends = 0.02). Higher maternal lutein concentrations were associated with a lower likelihood of poor VA in children (RR 0.60 (95% CI: 0.40, 0.88) for middle tertile; RR 0.78 (95% CI: 0.51, 1.19) for highest tertile (*p*-Quadratic = 0.02)). In conclusion, lutein and zeaxanthin status during pregnancy may influence offspring early visual development; but the results require confirmation with further studies, including more comprehensive measurements of macular functions.

## 1. Introduction

The macula is an oval and pigmented structure in the centre of human retina responsible for colour discrimination, motion detection, contrast sensitivity, and acuity [1]. High visual acuity (VA) refers to the ability to see fine detail, which is dependent on accurate refraction of light on the retina, a functioning visual system, and an intact structure of the macula [1]. Ensuring optimal macular health may therefore contribute to better VA. 

The macula uniquely concentrates two carotenoids—lutein and zeaxanthin—which protect the macula from light-related oxidative damage [2]; and may be important for macular health as well as facilitating good visual performance. Lutein and zeaxanthin have been implicated in visual function in older adults and shown to protect against age-related macular degeneration (AMD). This is supported by clear evidence from trials showing that lutein and zeaxanthin supplementation improve visual functions (e.g., acuity, contrast sensitivity) in older adults with AMD [3]. 

Deposition of lutein and zeaxanthin in the macula occurs as early as foetal life [2]. Lutein and zeaxanthin have been detected in the macula around 14 weeks of gestation and continues to increase in deposition until early childhood [4,5,6], corresponding to the development of key retinal structures and visual functions [7]. The timing suggests that lutein and zeaxanthin may influence the early development of the retinal system including the macula, or protect the developing retina/macula from oxidative damage [8,9], which results from exposure to the pro-oxidant in utero environment required for normal foetal development [10].

Limited studies have examined the role of lutein and zeaxanthin in early retinal/macular development in humans, but evidence from animal studies suggest that a lack of lutein and zeaxanthin since birth can produce abnormalities in the retinal pigment epithelium [11,12]. One study in preterm infants found those supplemented with lutein and zeaxanthin to have greater rod photoreceptor sensitivity—indicative of better visual performance when ambient illumination is low [13]. Additional evidence to support the role of lutein and zeaxanthin in retinal development comes from studies demonstrating better visual performance in infants who were breastfed compared to those formula-fed [14,15], which could be due to lutein and zeaxanthin being more abundant and bioavailable in breast milk than those in infant formulas [16].

To the best of our knowledge, no studies have examined whether exposures to these carotenoids during the in utero period represent a critical/sensitive window for optimal macular health. While studies have shown maternal lutein and zeaxanthin concentrations, whether from serum post-delivery or breast milk, to correlate with offspring macular pigment optical density (MPOD) [14,17] —an optical indicator of lutein and zeaxanthin concentrations [18]—they have not related maternal lutein and zeaxanthin to offspring visual functions. This study sought to relate plasma concentrations of maternal lutein and zeaxanthin during pregnancy to VA (a proxy of macular health) in 3-year old children.

## 2. Materials and Methods 

### 2.1. Study Sample

The Growing Up in Singapore Towards healthy Outcomes (GUSTO) study is a mother–offspring prospective cohort. A detailed cohort description has been previously published [19]. In brief, pregnant women (>18 years, <14 weeks gestation) from National University Hospital and KK Women’s and Children’s Hospital were invited to participate from June 2009 to September 2010. Chinese, Malay, and Indian women, whose parents and whose spouses’ parents were of homogenous ethnicity, were eligible to participate. Additional inclusion criteria included having the intention to deliver in either hospital, a plan to reside for the next five years in Singapore, and a willingness to donate birth tissues at delivery. Women with serious health conditions or diagnosed to have type-1 diabetes were excluded. The GUSTO study received ethics approval by the institutional review boards of both hospitals, and all procedures were according to the Declaration of Helsinki. Written informed consent were obtained at all study visits. 

**Figure 1:**
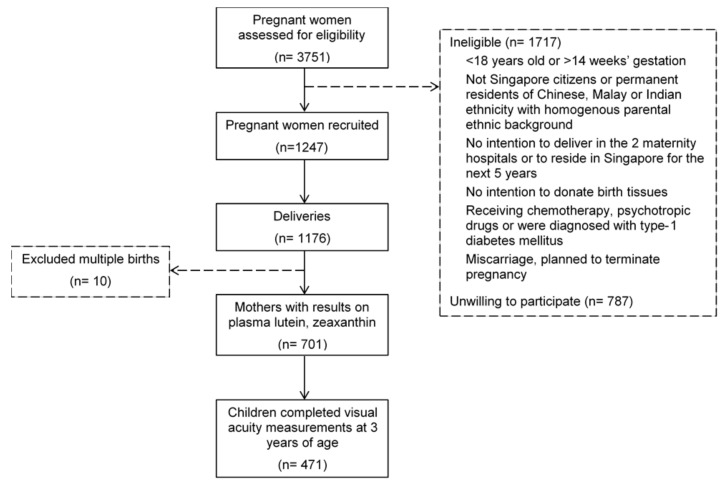
Flowchart of participants selected for analysis of associations of maternal plasma lutein and zeaxanthin with offspring visual acuity in the Growing Up in Singapore Towards healthy Outcomes cohort.

The present analysis included mothers who had plasma carotenoids concentrations measured at delivery, and their offspring completed VA measurement at age 3 years (*n* = 471; Figure 1). These mothers tended to be older, of Chinese ethnicity, and to have attained higher education (Supplementary Table S1), compared to mothers not included in the analysis.

### 2.2. Maternal Plasma Lutein and Zeaxanthin

Non-fasting bloods samples were obtained from mothers during delivery using the venipuncture technique. Ultra-High Performance Liquid Chromatography (UPLC) with Photo-Diode Array detection method quantified lutein and zeaxanthin concentrations in plasma. The UPLC is a special variant of a previously established HPLC method [20], but using columns with particle sizes less than 2.6 µm to allow for better separation and faster analysis. The precision of the method was similar to what has been previously published, and the inter- and intra-assays were <10% and <15%, respectively [20].

### 2.3. Children’s Eye Measurements at Age 3 Years

Children’s eye measurements were performed at age 3 years. Only one child had corrected distance VA measured, thus the current analysis is based on uncorrected distance VA measured in both eyes of the children using the LEA Symbols chart at a viewing distance of 3 m [21]. The line where at least three of four symbols were named correctly was taken as the VA value, expressed in the equivalent 6 metres VA conversion (6/x), which was directly read off the chart [21]. The readings were converted to the logarithm of the minimum angle of resolution (logMAR)—higher values indicate poorer VA while lower values indicate better VA. Poor VA was defined as >0.3 logMAR, with normal VA as ≤0.3 logMAR [22]. VA readings between right and left eyes were highly correlated (*p* = 0.80); only data from the right eye was used. 

Cycloplegic autorefraction measurements were also performed to assess refractive errors such as myopia, hyperopia, and astigmatism [23,24]. First, cycloplegia was accomplished after instillation of 0.5% proparacaine, 2.5% phenylephrine, and three drops of 1% cyclopentolate with each drop at 5-min intervals. Thirty-minutes after the last drop instillation, refraction readings (spherical, cylinder, and axis) were obtained using Canon RK-1 autorefractor (Tokyo, Japan). Spherical equivalent (SE) was sphere plus half cylinder power [23,24]. Myopia was defined as SE ≤ −0.5 dioptres, hyperopia as SE ≥ +3.00 dioptres, and astigmatism as cylinder ≤ −1.50 dioptres [25]. 

### 2.4. Covariates

Information on maternal age, ethnicity, and the highest education attained were collected during recruitment or the first GUSTO study visit. Mothers’ breastfeeding practices were obtained by trained interviewers during postnatal visits at 3 weeks, then at 3-month intervals up until 12 months. Parental myopia status was obtained at the 2-year postnatal visit and defined as either or both parents wearing or used to wear glasses or contact lenses for distant or near viewing. Children’s diets, including fruit and vegetable intakes, were assessed using a 24-h recall administered by trained research staffs. 

### 2.5. Statistical Analyses

Chi-square tests (categorical variables), independent samples t-test or Wilcoxon rank sum test (continuous variables with normal or skewed distributions) compared participant characteristics according to VA status. 

Associations of maternal lutein and zeaxanthin with logMAR VA were examined using linear regressions; and with poor VA using Poisson regression with robust error variance to estimate relative risk. 

Maternal plasma carotenoid concentrations were first modelled as tertiles (lowest tertile as reference) and tested for linear trends; then as continuous with a quadratic term to identify non-linear relationship. 

Statistical models were adjusted for child’s sex and exact age at eye examination; as well as for maternal age, ethnicity, education, and parental myopia (Model 1). Additional adjustment for breastfeeding duration in the first 12 months and child’s fruit and vegetables intake at age 3 years was performed, to determine if the associations between maternal carotenoids and child VA can be explained by postnatal nutritional factors (Model 2). 

To eliminate the influence of refractive errors, a common cause of reduced VA in children [22,25], we further adjusted for spherical refraction or SE (Model 3); and as a sensitivity analysis removing children with myopia, hyperopia, or astigmatism. 

We imputed missing values for covariates 20 times using multiple imputation technique with chained equations. All analyses were performed using Stata 14 (StataCorp, TX, USA). We considered two-sided *p* < 0.05 to be statistically significant.

## 3. Results

### 3.1. Characteristics of Mother–Offspring Pairs

We found 126 children to have poor VA, and 58 children to have refractive errors (30 had both poor VA and refractive error while 28 had refractive error but were recorded as having normal VA). No differences in maternal and child characteristics were observed between those with normal or poor VA (Table 1). 

### 3.2. Maternal Lutein and Zeaxanthin with Child Visual Acuity at Age 3 Years

The associations of maternal plasma lutein and zeaxanthin concentrations with children’s visual acuity at age 3 years are presented in Table 2.

After adjustment for confounders (Model 1), the highest tertile of maternal zeaxanthin concentrations was associated with 37% lower risk of children having poor VA (95% CI: 0.43, 0.95; Table 2A) in a dose-response manner (*p*-Trend = 0.03). In keeping with this, the highest tertile was associated with 0.03 lower logMAR (95% CI: −0.06, −0.003; *p*-Trend = 0.03; Table 2B). 

Higher maternal lutein concentrations were associated with 38% (95% CI: 0.42, 0.91) and 27% (95% CI: 0.48, 1.11) lower likelihood of poor VA in children, for the middle and highest tertile, respectively (*p*-Quadratic = 0.02; Table 2A). Likewise, a 0.04 (95% CI: −0.07, −0.003) and 0.01 (95%CI: −0.04, 0.03) lower logMAR in children, for the middle and highest tertile, respectively (*p*-Quadratic = 0.03; Table 2B).

The above associations remained statistically significant after further adjustment for breastfeeding duration and child’s fruit and vegetable intake (Model 2), as well as for spherical refraction (Model 3). 

After removing all children with myopia, hyperopia, and astigmatism (*n* = 58), the associations were in similar directions but did not reach statistical significance (Table 3).

## 4. Discussion

The present study observed higher maternal zeaxanthin concentrations to be associated with a lower risk of poor VA in children, and a U-shaped association between maternal lutein concentrations and child VA. 

Our finding of a significant positive association between maternal zeaxanthin and children’s VA is reminiscent of evidence in older adults showing improvements in VA following lutein and zeaxanthin supplementation [3], although the pathways of macular degeneration differ from those of visual development. The effect estimates were also comparable. We observed a 0.03 lower logMAR in children for the highest versus the lowest concentrations of maternal zeaxanthin, while the meta-analysis of trials in older adults reported a pooled mean difference of 0.04 reduction in logMAR in the lutein and zeaxanthin supplemented group compared to the placebo group [3]. 

The observed U-shaped association between maternal lutein and child’s VA could be interpreted as lutein being required in lesser amounts than zeaxanthin for optimal macular development, as the effect sizes were somewhat larger and significant for the middle tertile instead of the highest. Observations from anatomical studies found zeaxanthin to be the dominant carotenoid in the fovea (the area in macula that confers the highest visual acuity), and also exists in greater amounts than lutein in individuals aged 3 years and above [6]. Alternatively, one study found maternal zeaxanthin concentrations but not lutein to correlate with infant MPOD [17], suggesting that maternal zeaxanthin may play a more important role than lutein in macular development during the in utero period. However, no studies have investigated the effects of different levels of lutein and zeaxanthin on visual performance or showed anatomical and functional differences in the macula to confirm our speculation. Understanding the importance of a balanced zeaxanthin:lutein ratio for macular development, and whether maternal lutein and offspring VA truly reflects a U-shaped association, requires further investigation. 

Much evidence supports a role for lutein and zeaxanthin in visual functions amongst older adults, but beneficial effects of these carotenoids on macular health may manifest in early life [2,26]. Assessments of lutein and zeaxanthin status at the end of life may result in a missed opportunity to identify the critical periods, sensitive periods, and cumulative effects of the relationship of lutein and zeaxanthin with macular health later in life. Our study provides novel data (albeit early evidence) on potential in utero influences of lutein and zeaxanthin on visual functions of the developing macula, but whether exposures to these carotenoids during the in utero period represent a critical/sensitive window for optimal macular health in the life course will require examination in longer term studies.

These findings, however, need cautious interpretation due to the many factors affecting VA in children. Our results were no longer statistically significant in the sensitivity analysis, suggesting possible confounding by refractive errors. Interestingly, the adjustment for spherical refraction did not attenuate our main findings, hence the loss of statistical significance could be a result of a smaller sample size. To date, there is no clear establishment of a relationship between lutein and zeaxanthin and refractive errors. Most studies found no differences in MPOD in subjects with different axial lengths and refractive errors. Given that the effect estimates were similar, the reduction in statistical power is likely the reason for non-statistical significance. Nonetheless, we acknowledged that if a child is inattentive or uncooperative during eye examinations, this can also result in low VA recordings, leading to misclassification bias. This misclassification bias, however, is likely to be non-differential, thus shifting the association towards null. Additionally, a poor VA status may be reflective of an immature, developing visual system that has yet to reach adult stage, rather than a true influence of nutritional exposures. 

Compared to past studies which have related maternal lutein and zeaxanthin to offspring vision, our study has several strengths: (1) Maternal lutein and zeaxanthin concentrations were measured immediately prior to delivery which is a better indicator of concentrations during pregnancy compared to measurements in breast milk; (2) this is the first study that examines the associations of these carotenoids with visual performance (i.e., visual acuity) in young children. The Lea Symbols chart used to assess VA is age-appropriate and requires only matching a card with the correct symbol rather than use of letters which requires literacy. 

This study is limited by having VA measures for only a subset of the children, which could lead to selection bias, but the characteristics that differed between those included vs. those excluded were adjusted for in our statistical models. By having only one measure of macular function limits the clinical relevance of our study findings—including the contrast sensitivity test could help confirm whether maternal lutein and zeaxanthin have influences on offspring visual performance. Having measurements of children’s MPOD at birth could also help confirm whether deposition of macular pigment is a result of greater exposures in utero. The measurement of maternal carotenoids at one time-point may not be an accurate reflection of concentrations over the entire pregnancy, but studies have shown minimal changes in dietary patterns throughout pregnancy [27,28], as well as no significant changes in plasma lutein and zeaxanthin concentrations from second to third trimesters [29,30] when deposition of macular pigment begins. We recognized that a child’s VA could also be influenced by their own nutritional status and have accounted for several postnatal nutritional factors, but this study could benefit from having measured children’s lutein and zeaxanthin concentrations. A number of important predictors of child myopia, such as light exposure or time spent outdoors [31,32], which could have confounded the associations observed, were not adjusted for, as their influence on macular health is unclear. 

## 5. Conclusions

Maternal lutein and zeaxanthin during pregnancy may play important roles in the visual developmental trajectory of the offspring. Our findings further add to current evidence on the beneficial effects of consuming sufficient quantities of dark green and orange coloured fruits and vegetables—key food sources of zeaxanthin and lutein during pregnancy for offspring health. However, the clinical implications for children’s visual health requires further investigation in longer term studies with larger samples sizes and more comprehensive eye measurements.

## Figures and Tables

**Table 1:** Maternal and infant characteristics according to visual acuity status of 3-year old children in Growing Up in Singapore Towards healthy Outcomes (GUSTO) (*n* = 471) ^1^.

	Low Visual Acuity	Normal Visual Acuity	
	>0.3 logMAR (*n* = 126)	≤0.3 logMAR (*n* = 345)	*p* ^2^
Maternal Characteristics			
Age (year), mean ± SD	30.8 ± 5.0	30.9 ± 5.2	0.76
Ethnicity, *n* (%)			
Chinese	71 (56)	208 (60)	0.30
Malay	28 (22)	84 (24)	
Indian	27 (22)	53 (16)	
Highest education, *n* (%)			
≤Secondary	42 (33)	95 (28)	0.30
Post-secondary	36 (29)	122 (36)	
University	48 (38)	126 (37)	
Parental myopia, *n* (%)			
Yes	98 (80)	270 (83)	0.58
No	24 (20)	57 (17)	
**Child Characteristics**			
Age at eye examination (month), mean ± SD	36.5 ± 1.1	36.4 ± 1.0	0.30
Sex, *n* (%)			
Male	68 (54)	174 (50)	0.50
Female	58 (46)	171 (50)	
Any breastfeeding duration, *n* (%)			
<1 month	29 (24.0)	74 (22.6)	0.91
1 to <3 months	21 (17.4)	62 (18.9)	
3 to <6 months	20 (16.5)	55 (16.8)	
6 to <12 months	25 (20.7)	57 (17.4)	
≥12 months	26 (21.5)	80 (24.4)	
Fruit and vegetables intake age 3 years (g/day), median (IQR)	69.8 (30.5, 143.3)	72.6 (21.6, 138.9)	0.76

GUSTO, Growing Up in Singapore Towards healthy Outcomes; IQR, inter-quartile range; logMAR, logarithm of the minimum angle of resolution; ^1^ Missing data: *n* = 2 maternal education and *n* = 22 parental myopia, *n* = 22 breastfeeding duration, *n* = 36 fruit and vegetables intake; ^2^ *p* values are for chi-square test (for categorical variable), independent samples t-test, or Wilcoxon rank sum test (for continuous variables with normal or skewed distributions).

**Table 2:** Associations of maternal lutein and zeaxanthin with poor visual acuity (**A**) and logMAR visual acuity (**B**) in GUSTO children (*n* = 471).

**A. Poor Visual Acuity (Defined as >0.3 logMAR)**			
	**Model 1 ^1^**	**Model 2 ^2^**	**Model 3 ^3^**
	**RR (95% CI)**	**RR (95% CI)**	**RR (95% CI)**
**Zeaxanthin Tertiles (Median; IQR µmol/L)**			
Tertile 1 (0.06; 0.05, 0.07)	(reference)	(reference)	(reference)
Tertile 2 (0.09; 0.08, 0.10)	0.91 (0.65, 1.29)	0.89 (0.63, 1.25)	0.89 (0.63, 1.25)
Tertile 3 (0.13; 0.12, 0.15)	0.63 (0.43, 0.95) *	0.62 (0.41, 0.92) *	0.62 (0.42, 0.93) *
*p*-Trends	0.03 *	0.02 *	0.02 **
*p*-Quadratic	0.52	0.41	0.43
Lutein Tertiles (Median; IQR µmol/L)			
Tertile 1 (0.08; 0.06, 0.09)	(reference)	(reference)	(reference)
Tertile 2 (0.13; 0.12, 0.15)	0.62 (0.42, 0.91) *	0.60 (0.40, 0.88) *	0.60 (0.40, 0.88) *
Tertile 3 (0.22; 0.18, 0.28)	0.73 (0.48, 1.11)	0.78 (0.51, 1.19)	0.78 (0.51, 1.19)
*p*-Trends	0.16	0.31	0.31
*p*-Quadratic	0.02 *	0.02 *	0.02 *
**B. LogMAR Visual Acuity**			
	**Model 1 ^1^**	**Model 2 ^2^**	**Model 3 ^3^**
	**β (95% CI)**	**β (95% CI)**	**β (95% CI)**
**Zeaxanthin Tertiles (Median; IQR µmol/L)**			
Tertile 1 (0.06; 0.05, 0.07)	(reference)	(reference)	(reference)
Tertile 2 (0.09; 0.08, 0.10)	−0.02 (−0.05, 0.01)	−0.02 (−0.05, 0.01)	−0.02 (−0.05, 0.01)
Tertile 3 (0.13; 0.12, 0.15)	−0.03 (−0.06, −0.003) *	−0.03 (−0.06, −0.001) *	−0.03 (−0.06, −0.001) *
*p*-Trends	0.03 *	0.04 *	0.04 *
*p*-Quadratic	0.97	0.92	0.91
Lutein Tertiles (Median; IQR µmol/L)			
Tertile 1 (0.08; 0.06, 0.09)	(reference)	(reference)	(reference)
Tertile 2 (0.13; 0.12, 0.15)	−0.04 (−0.07, −0.003) *	−0.04 (−0.07, −0.01) *	−0.04 (−0.07, −0.01) *
Tertile 3 (0.22; 0.18, 0.28)	−0.01 (−0.04, 0.03)	−0.01 (−0.04, 0.03)	−0.01 (−0.04, 0.03)
*p*-Trends	0.79	0.85	0.85
*p*-Quadratic	0.03 *	0.04 *	0.04 *

GUSTO, Growing Up in Singapore Towards healthy Outcomes; IQR, inter-quartile range; logMAR, logarithm of the minimum angle of resolution, ^1^ Model 1: Adjusted for child’s age at eye measurement and sex; maternal age, ethnicity, education; and parental myopia, ^2^ Model 2: Model 1 + breastfeeding duration, child’s fruit and vegetable intake at 3 years, ^3^ Model 3: Model 2 + spherical refraction, * *p* < 0.05.

**Table 3:** Associations of maternal lutein and zeaxanthin with poor visual acuity (**A**) and logMAR visual acuity (**B**) in GUSTO children without myopia, hyperopia, and astigmatism (*n* = 413).

**A. Poor Visual Acuity (Defined as >0.3 logMAR)**			
	**Model 1** ^1^	**Model 2** ^2^	**Model 3** ^3^
	**RR (95% CI)**	**RR (95% CI)**	**RR (95% CI)**
**Zeaxanthin Tertiles (Median; IQR µmol/L)**			
Tertile 1 (0.06; 0.05, 0.06)	(reference)	(reference)	(reference)
Tertile 2 (0.09; 0.08, 0.10)	0.97 (0.65, 1.46)	0.98 (0.65, 1.47)	0.98 (0.65, 1.46)
Tertile 3 (0.13; 0.12, 0.16)	0.73 (0.45, 1.17)	0.72 (0.45, 1.16)	0.73 (0.45, 1.18)
*p*-Trends	0.21	0.19	0.20
*p*-Quadratic	0.77	0.67	0.62
Lutein Tertiles (Median; IQR µmol/L)			
Tertile 1 (0.08; 0.06, 0.09)	(reference)	(reference)	(reference)
Tertile 2 (0.13; 0.12, 0.15)	0.68 (0.44, 1.07)	0.69 (0.44, 1.08)	0.69 (0.44, 1.08)
Tertile 3 (0.22; 0.18, 0.26)	0.85 (0.52, 1.39)	0.86 (0.53, 1.42)	0.87 (0.53, 1.43)
*p*-Trends	0.29	0.67	0.69
*p*-Quadratic	0.05	0.12	0.12
**B. LogMAR Visual Acuity**			
	**Model 1** ^1^	**Model 2** ^2^	**Model 3** ^3^
	**β (95% CI)**	**β (95% CI)**	**β (95% CI)**
**Zeaxanthin Tertiles (Median; IQR µmol/L)**			
Tertile 1 (0.06; 0.05, 0.06)	(reference)	(reference)	(reference)
Tertile 2 (0.09; 0.08, 0.10)	−0.01 (−0.04, 0.02)	−0.01 (−0.04, 0.02)	−0.01 (−0.04, 0.02)
Tertile 3 (0.13; 0.12, 0.16)	−0.02 (−0.05, 0.02)	−0.02 (−0.05, 0.02)	−0.02 (−0.05, 0.02)
*p*-Trends	0.34	0.30	0.31
*p*-Quadratic	0.95	0.99	0.92
Lutein Tertiles (Median; IQR µmol/L)			
Tertile 1 (0.08; 0.06, 0.09)	(reference)	(reference)	(reference)
Tertile 2 (0.13; 0.12, 0.15)	−0.02 (−0.05, 0.02)	−0.02 (−0.05, 0.02)	−0.02 (−0.05, 0.02)
Tertile 3 (0.22; 0.18, 0.26)	0.01 (−0.03, 0.05)	0.01 (−0.02, 0.05)	0.02 (−0.02, 0.05)
*p*-Trends	0.56	0.36	0.32
*p*-Quadratic	0.16	0.13	0.13

GUSTO, Growing Up in Singapore Towards healthy Outcomes; IQR, inter-quartile range; logMAR, logarithm of the minimum angle of resolution, ^1^ Model 1: Adjusted for child’s age at eye measurement and sex; maternal age, ethnicity, education; and parental myopia, ^2^ Model 2: Model 1 + breastfeeding duration, child’s fruit and vegetable intake at 3 years, ^3^ Model 3: Model 2 + spherical refraction.

## Supplementary Materials

The following are available online at https://www.mdpi.com/2072-6643/12/2/274/s1, Table S1: Characteristics of included versus excluded mother-child pairs for analysis of maternal plasma lutein and zeaxanthin concentrations and child visual acuity at age 3 years in the Growing Up in Singapore Towards healthy Outcomes study.
