# Supplementary material for: Maternal Lutein and Zeaxanthin Concentrations in Relation to Offspring Visual Acuity at 3 Years of Age: The GUSTO Study
Source: Nutrients. 2020 Jan 21;12(2):274. doi: 10.3390/nu12020274 (PMC7070638; doi:10.3390/nu12020274)
Supplement: Supplementary file 1 [file nutrients-12-00274-s001.pdf]

Supplementary Table S1: Characteristics of included versus excluded mother-child pairs for analysis of maternal plasma lutein and zeaxanthin concentrations and child visual acuity at age 3 years in the Growing Up in Singapore Towards healthy Outcomes study[†, ‡].

	**Included**	**Excluded**	***P*[c]**
	n = 471	n = 617	
**Maternal Characteristics**			
Age (year), mean ± SD	31.5 ± 5.1	30.6 ± 5.1	0.006*
Ethnicity, *n* (%)			
Chinese	279 (59.2)	317 (51.4)	0.035*
Malay	112 (23.8)	178 (28.8)	
Indian	80 (17.0)	122 (19.8)	
Highest education, *n* (%)			
≤Secondary	138 (29.4)	202 (33.4)	0.034*
Post-secondary	157 (33.5)	224 (37.0)	
University	174 (37.1)	179 (29.6)	
Parental myopia (yes), *n* (%)	366 (81.3)	321 (76.3)	0.066
**Child Characteristics**			
Sex, *n* (%)			
Male	242 (51.4)	329 (53.3)	0.525
Female	229 (48.6)	288 (46.7)	
Any breastfeeding duration, *n* (%)			
<1 month	103 (22.9)	151 (29.5)	0.077
1 to <3 months	83 (18.5)	102 (20.0)	
3 to <6 months	75 (16.7)	84 (16.4)	
6 to <12 months	82 (18.3)	81 (15.8)	
≥12 months	106 (23.6)	93 (18.2)	
Fruit and vegetables intake at 3 years (g/day), median (IQR)	70.7 (22.8, 142.2)	79.3 (24.2, 137.8)	0.823

[†] Missing data: n=14 maternal education, n = 271 parental myopia, n=128 breastfeeding duration, n=297 child's fruit and vegetables intake

[‡] *P*-values (*P<0.05) were obtained from chi-square test; independent t-test or Wilcoxon rank-sum tests.
